# Lower Extremity Stretch-Shortening Cycle Performance in the Vertical and Horizontal Direction as Key Determinants of Success in Collegiate Male Taekwondo

**DOI:** 10.3390/jfmk10010015

**Published:** 2025-01-02

**Authors:** Chieh-Ying Chiang, Yi-Chien Chiang, Hsuan-Yu Lin, Hao-Che Tseng, Mu-Yen Chu, Jung-San Chang

**Affiliations:** 1 Department of Sports Training Science-Combats, National Taiwan Sport University, Taoyuan City 333, Taiwan; 2 Taiwan Institute of Sports Science, Kaohsiung City 813, Taiwan; 3 Graduate Institute of Athletics and Coaching Science, National Taiwan Sport University, Taoyuan City 333, Taiwan; 1120321@ntsu.edu.tw (Y.-C.C.); hsuanyu1997@gmail.com (H.-Y.L.); tsengbase@gmail.com (H.-C.T.)

**Keywords:** countermovement jump, leg power, reactive strength, dynamic strength

## Abstract

Background/Objectives: The underlying mechanisms of taekwondo-specific jumping ability among different competition levels are still unknown. This study aimed to compare vertical and horizontal stretch-shortening cycle (SSC) performance between athletes of different competitive levels and examine the relationships of force and power production abilities between those two directions in Taiwanese collegiate-level male taekwondo athletes. Methods: Seventeen male collegiate taekwondo athletes were divided into two groups: medalists (MG, n = 8) and non-medalists (NMG, n = 9); both groups performed countermovement jumps (CMJ) on a force platform and single-leg lateral hops (SLLHs) via an optoelectronic measurement system. Eccentric and concentric phase measures from CMJ and distant and temporal variables of SLLH were collected for further analysis. Results: The MG achieved statistically superior jump height (JH), concentric peak velocity, eccentric peak force, force at 0 velocity, and eccentric displacement than NMG (*d* = 1.05–1.36). Although non-significant differences showed in SLLH variables, MG had better results than NMG (*d* = 0.40–0.84). Moderate relationships were identified between SLLH step 1 and JH, reactive strength index modified, peak concentric power, and peak concentric velocity of CMJ (*r* = 0.50–0.57, *p* < 0.05). Furthermore, step 2 ground contact time and lateral reactive strength index from SLLH were associated with CMJ peak eccentric force, peak concentric force, and force at 0 velocity (*r* = 0.53–0.59, *p* < 0.05). Conclusions: Taekwondo MG achieved superior CMJ and SLLH performance. In addition, jumping abilities in vertical and lateral directions shared specific underlying mechanisms in collegiate male taekwondo athletes.

## 1. Introduction

Taekwondo is a full-contact combat sport involving punching and kicking, yet lower extremities techniques are more predominant in training and competitions [1,2]. Therefore, high muscular power in the lower limbs is beneficial for fighters to execute the stretch-shortening cycle (SSC) muscle actions and perform many sequences of aggressive attacks and counterattacks [3]. In addition, evidence has indicated that jumping ability is a crucial physical attribute in evaluating lower-body SSC and can differentiate the more successful from less successful taekwondo athletes [2].

Among jumping tests, the countermovement jump (CMJ) has been implemented in previous studies to assess performance in taekwondo athletes [2,4,5,6,7,8,9,10]. In these studies, the comparisons of CMJ performance between different levels of athletes showed similar results, concluding jump height (JH) to be a discriminative variable between the experienced and novice [10], the elite and recreational [8], medalists and non-medalists [2], the national-selected and non-selected [6], and senior and junior [8]. However, JH alone may not provide enough detailed information on the muscular power characteristics of taekwondo athletes. Furthermore, the unilateral moving nature of taekwondo’s attacking and defending skills may require additional assessment of the SSC performance in the horizontal direction [1,2]. Based on the current evidence, further research is needed to better understand both the vertical and horizontal direction SSC mechanisms contributing to superior performance in taekwondo athletes.

Recently, using the force plate to assess athletes’ CMJ performance is considered the gold standard due to its versatility in measuring various kinematic and kinetic variables [11]. The ground reaction force (GRF) recorded through the CMJ tests can be further tailored to force–time, velocity–time, power–time, and displacement–time curves [12,13,14,15]. Thus, JH, time to take off (TTT), eccentric and concentric time and displacement, reactive strength index modified (RSImod), eccentric and concentric peak force, peak power, and peak velocity [13,15,16], and force at 0 velocity (F@0V) can be analyzed from the abovementioned curves [15,17]. Furthermore, previous studies suggested that the comparisons across these variables can reveal different neuromuscular statuses between the higher and lower levels of sprinters [18], rugby league players [15], and mixed martial arts (MMA) competitors [16]. However, to our knowledge, no study has conducted an in-depth analysis of CMJ performance in taekwondo athletes to understand better the underpinning mechanisms to discriminate taekwondo athletes at different competitive levels. Also, the CMJ primarily assesses vertical jump performance and therefore may not provide a complete picture of the neuromuscular function required for the multi-planar movements inherent to taekwondo.

Taekwondo kicking techniques are predominantly performed unilaterally and mostly in the frontal plane, characterized by a lateral fighting stance to move unilaterally in the horizontal direction [1,2,19,20]. Several studies have suggested the benefit of using the triple hop for distance (THD) to assess unilateral leg power [21,22]. The hop test includes executing more than one step undergoing a plyometric-type action to overcome an eccentric loading produced by the initial jump and immediately perform a concentric contraction to finish the task—requiring reactive forces from SSC [21]. This type of movement could resemble taekwondo, which is unilateral and under high stretch-shortening cycle loads [3]. However, the typical hop test performed is in the sagittal plane. Recently, a novel test, single-leg lateral hop (SLLH), has been developed to test athletes’ lateral jumping ability [23,24]. Unlike the THD, the SLLH requires horizontal power in the lateral direction, with both SSC and reactive muscle contractions involved. Moreover, how taekwondo athletes of different levels perform in this test is still unclear.

Despite the importance of both vertical and lateral SSC function in taekwondo, it remains unclear whether these two capacities can differentiate taekwondo athletes of varying skill levels. Furthermore, no research has specifically examined the relationship between CMJ and SLLH performance. This study aims to address this gap by examining the CMJ and SLLH characteristics of Taiwanese collegiate male taekwondo athletes with varying competitive success. Specifically, this study aims to: (1) compare CMJ and SLLH performance between more successful and less successful taekwondo athletes and (2) investigate the relationships of force and power production abilities between CMJ and SLLH tests.

## 2. Materials and Methods

### 2.1. Experimental Approach to the Problem

A group of collegiate-level taekwondo athletes was divided into the medalist group (MG) and non-medalist group (NMG) based on their winning records achieved at national-level competitions within 6 months before this study. All participants were tested during the competitive phase, in the same period (2019–2020 season). They were instructed to avoid any strenuous physical activity in the 24 h before testing. In addition, all data included in this study were collected as part of an ongoing athlete performance monitoring program, and all the athletes involved were familiar with the testing procedures.

### 2.2. Participants

Seventeen male taekwondo athletes participated in this study. The MG (n = 8; age, 20.2 ± 1.0 years; height, 179.5 ± 4.5 cm; weight, 71.7 ± 9.7 kg) consisted of medalist athletes with top 3 winning records from competitions, while the NMG (n = 9; age, 19.8 ± 1.0 years; height, 173.3 ± 5.8 cm; weight, 69.0 ± 8.7 kg) were those with non-winning records. They were members of the National Taiwan Sport University taekwondo team with over 6 years of training experience and a minimum training age of 1 year in a structured strength and conditioning program. None were involved in intensive weight-loss practices during the selected period, and all the athletes were free from any injury or neuromuscular disorder. The Institutional Review Board (IRB) at Fu Jen Catholic University approved the investigation, and written informed consent was obtained from the subjects before study participation. 

The two tests were completed on a single occasion in a randomized order. First, all subjects had their anthropometric qualities (height in centimeters and body mass in kilograms to the nearest 0.1 kg and 0.1 cm, respectively) evaluated with a stadiometer (BMS370; Inbody Corporation, Seoul, Republic of Korea). Subsequently, each subject completed a 15-min standardized warm-up, including prescribed dynamic stretching and practice jumps led by the same research team member. 

### 2.3. Procedures

#### 2.3.1. Countermovement Jump

Each subject was set up for the CMJ in a standing position with a dowel placed across the shoulders. The instruction was to sink to a self-selected depth as quickly as possible and jump as high as possible with legs fully extended during the flight phase. A minimum of two maximal effort trials was required for data collection. A CMJ warmup attempt was given before performing two maximal effort trials, with one minute of recovery period allocated between each trial. CMJs that were not consistent (jump height within 2 cm of a previous trial qualified as consistent) or inadvertently performed with the inclusion of leg tuck during the flight phase were discarded and repeated after rest. A limitation of four attempts was set for this study for safety concerns, and the research team visually monitored each attempt to identify mistrials. 

The CMJs were performed with the subjects standing on a force platform (2812A, Kistler., Winterthur, Switzerland) sampling at 1000 Hz with an analog-to-digital converter (2812A, Kistler., Winterthur, Switzerland) interfaced to the platform for data acquisition and signal processing using Bioware 5.0 software (2812A, Kistler., Winterthur, Switzerland). Raw vertical-force–time data were recorded and stored, subsequently exported as text files, and analyzed using a custom-designed Microsoft Excel spreadsheet (version 2016, Microsoft Inc., Redmond, WA, USA). 

**Figure 1:**
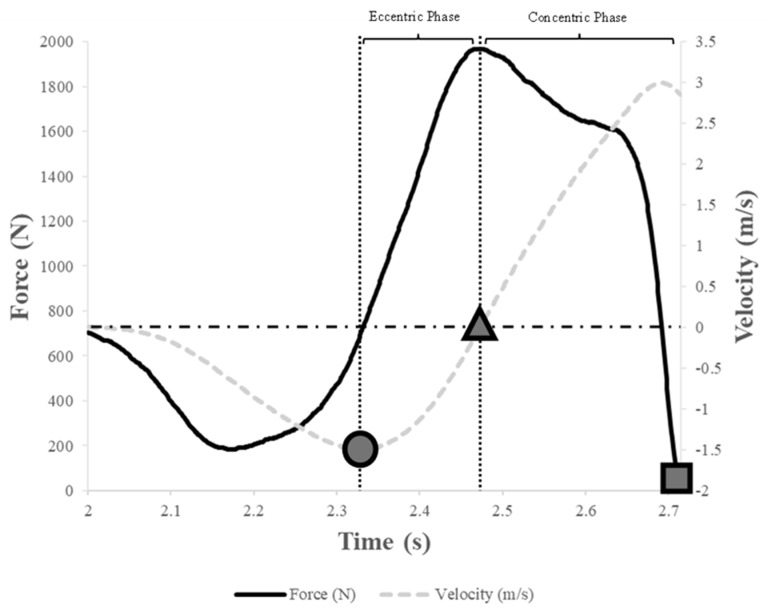
Illustration of the CMJ phase analysis methods. The black line represents force–time data. The gray dotted line represents the velocity–time data as the integral of vertical acceleration with respect to time. The grey circle is the timepoint where peak negative velocity is reached. The grey triangle is the timepoint where velocity crosses zero. The grey square is the timepoint where force fell below 5 times the SDs of the flight-phase force. The eccentric phase was defined as the time between peak negative velocity and zero velocity. The concentric phase was defined to have started when velocity exceeded 0.01 m/s and finished at take-off.

For subsequent data analyses, system weight was defined as the 1-second period when the subjects stood motionless before starting movement [25]. The onset threshold of each jump was determined by 5 times the standard deviation (SD) of the vertical ground reaction force across the first second. In line with previous recommendations [25], the onset of each jump was considered 30 ms before the instant where vertical force decreased below the calculated threshold to system weight. Vertical acceleration of the center of mass (COM) was computed using Newton’s Law of Acceleration (Force = Mass × Acceleration). COM velocity was calculated as the integral of vertical acceleration with respect to time using the trapezoidal rule. COM displacement was subsequently determined by integrating velocity data with respect to time [15]. Power was calculated by multiplying vertical force and velocity data at each time point. As shown in Figure 1, the eccentric phase of the CMJ was defined as the time between the instants of peak negative COM velocity and zero COM velocity. The concentric phase of the CMJ was deemed to have started when COM velocity exceeded 0.01 m/s and finished at take-off [13,25]. Take-off was identified when vertical force fell below 5 times the SDs of the flight-phase force [13,14,15]. Once each phase was identified, the custom spreadsheet was used to analyze key variables of the CMJ. Table 1 provides detailed descriptions of these variables and outlines the analysis methods employed. Force and power were expressed relative to body mass for further analysis.

#### 2.3.2. Single-Leg Lateral Hop

**Figure 2:**
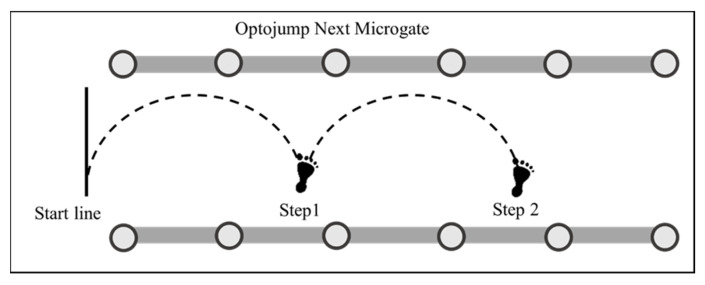
Illustration of the SLLH. Subjects perform two consecutive lateral hops. Step 1, step 2 distance (dot lines), and step 1 GCT were collected.

**Figure 3:**
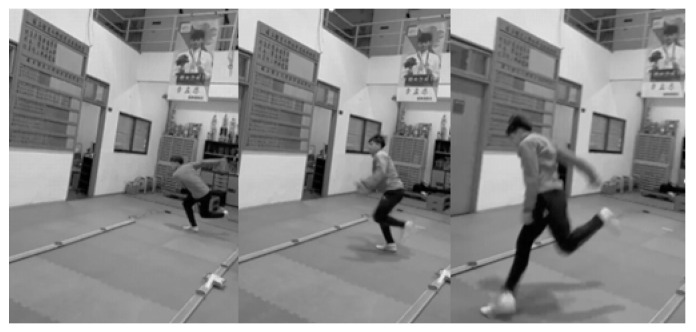
Visual example of the SLLH. Subjects were instructed to use a self-selected countermovement immediately followed by two consecutive single-leg lateral hops for maximal distance while minimizing ground contact time. Arm swings were allowed in the countermovement to assist the hop and maintain balance.

Each subject started by standing on the testing leg with the medial border of the foot level and the start line marked with the athletic tape. They were instructed to use a self-selected countermovement immediately followed by two consecutive single-leg lateral hops for maximal distance while minimizing ground contact time [24] (Figure 2). Arm swings were allowed in the countermovement to assist the hop and maintain balance. Figure 3 presents a visual demonstration of each phase of the test. A warm-up attempt was given for each leg before performing three maximal effort trials, with a one-minute rest between each test. All hops were completed with one leg before finishing with the other in a counterbalanced order. Trials were excluded and repeated if the subject did not land with the test leg or touch the floor with different body parts once the jump cycle had begun. The research team visually monitored each attempt to identify mistrials. Data for SLLH were collected using an optoelectronic measurement system (Optojump Next, Version 1.3.20.0, Microgate, Bolzano, Italy). This method has been validated for measuring contact times [26]. As shown in Figure 2, ground contact time (GCT) was extrapolated from ground contacts determined by the disruption of the infrared bar. GCT was defined as the period when the foot disrupted the optoelectronic system during landing and take-off. Step 1 was defined as the distance between the start line and the contact point of the first step, and step 2 was defined as the start line and the contact point of the second step. Lateral reactive strength index (LRSI) was calculated by dividing step 2 by GCT.

### 2.4. Statistical Analyses 

Data were analyzed with descriptive statistics, and results are summarized as mean ± SD. The mean output of the two CMJ trials and the best SLLH trial (with the most significant total distance among all hops) were taken for further statistical analysis [27]. The distribution of all the data was examined using the Shapiro–Wilk normality test. Between-trials reliability of the jumps was assessed using two-way mixed intraclass correlation coefficients (ICC) and coefficients of variation (CV). The ICC values were interpreted according to previous research where values ranging from 0.40 to 0.75 are considered good, and those over 0.75 are considered excellent [28]. A CV of ≤10% was considered to reflect acceptable variability in line with previous recommendations [29]. CMJ and SLLH parametric variables were compared using independent t-tests to examine between-group jumping performances. Nonparametric variables were compared via Mann–Whitney *U* test determined by the normality test. An alpha level of *p* ≤ 0.05 was set to identify statistical significance. In addition, the magnitude of differences was determined by using Cohen’s *d* effect size (ES) and are interpreted as trivial (<0.20), small (0.20–0.59), moderate (0.60–1.19), large (1.20–1.99), and very large (>2.00) [30]. Pearson’s product–moment correlations were used to analyze relationships between CMJ and SLLH performance. Correlation coefficients were interpreted as trivial (<0.10), small (0.10–0.29), moderate (0.30–0.49), large (0.5–0.69), very large (0.70–0.89), and nearly perfect (>0.9) [30]. All statistical analyses were conducted using SPSS Statistics 24 (IBM SPSS, Inc., Chicago, IL, USA).

## 3. Results

All CMJ and SLLH variables satisfied the normality test except for CMJ TTT. CMJ variables demonstrated good to excellent between-trial reliability and acceptable between-trial variability (Table 2). Significantly greater JH was attained by the MG (*p* = 0.01, *d =* 1.40) (Table 2). The MG also demonstrated greater relative values in EccPF (*p* = 0.04, *d =* 1.36), F@V0 (*p* = 0.04, *d* = 1.08), and COM displacement (*p* = 0.05, *d* = 1.05) during the eccentric phase of the jump. Meanwhile, in the CMJ concentric phase, only ConPV showed significantly greater performance in the MG. Between-trials reliability (ICC = 0.687–0.897) and variability (CV = 3.00–7.08) were also acceptable for SLLH. Although none of the variables reached statistical significance between the MG and the NG, the MG demonstrated greater values in all SLLH measures (*d* = 0.40–0.84) (Table 3). Correlation among the CMJ and SLLH variables is presented in Table 4. Note that moderate to large positive correlations with statistical significance exist between Step 1 and JH, Step 1 and RSImod, Step 1 and ConPP, Step 1 and ConPV, LRSI and EccPF, LRSI and ConPF, and LRSI and F@0V (*r =* 0.495–0.593, *p* = 0.01–0.04). Moderate to large negative correlations were found in CT and EccPF, CT and ConPF, and CT and F@0V (*r =* 0.532–0.555, *p* = 0.02–0.03), whereas the remaining variables showed low to moderate and no significant correlation. 

## 4. Discussion

This is the first study to examine the jumping mechanisms among taekwondo athletes with different competitive levels. We aimed to address the gap in existing research by examining the relationship between CMJ and SLLH performance in Taiwanese collegiate male taekwondo athletes. Specifically, we investigated whether differences in jump characteristics exist between more successful and less successful athletes and explored the relationships between CMJ and SLLH. The primary findings of this study are as follows. Based on the CMJ results, eccentric phase variables accounted for significant between-group differences. In addition, the MG reached greater values in all SLLH variables, indicating that the force production ability in the lateral direction may play an equally vital role in taekwondo performance. Finally, moderate to large correlations indicate that CMJ and SLLH share similar underlying neuromuscular mechanisms. 

A unique finding of this study was that the better eccentric phase characters might explain the superior CMJ performance in MG. In addition, significantly greater EccPF and F@0V suggested that the MG could better absorb and store the energy in series elastic elements generated after the pre-stretch for subsequent re-utilization in the concentric phase [31]. 

In addition, F@0V represents the forces that can be utilized to transition from the eccentric to concentric phase, suggesting the MG may have a superior ability to perform the braking before re-accelerating in a CMJ task. Taekwondo players need rapid change of direction abilities and mechanical efficiency to achieve optimal positions when moving in and out of offensive or defensive positions [3]. Thus, being able to accelerate and decelerate rapidly is required by the nature of taekwondo. A taekwondo athlete with greater eccentric force production capacity might have greater breaking strategies to choose from [17] and aid defensive actions by absorbing attacks [3], which facilitates quick stabilization for launching further counterattacks. However, these results are inconsistent with the previous study, which showed the eccentric strength characters did not differentiate mixed martial arts (MMA) fighters from different competition levels [16]. Thus, the eccentric strength characteristics variable could be representative of a crucial physical attribute in medalist athletes that potentially underpins taekwondo performance. 

Furthermore, this study discovered possible mechanics for greater JH in the MG. Superior eccentric performance in MG was characterized by greater countermovement without spending extra time (i.e., TTT) than the NMG. In a CMJ, employing deeper countermovement results in optimizing SSC mechanics [12]. It enables muscles to build up a high level of active state and force before the start of shortening, thereby allowing greater work at the end of the stretch and the first phase of muscle shortening, contributing to greater JH [32]. 

Applying a deeper countermovement that resulted in higher force generated during the initiation of muscle shortening might reflect similar mechanisms during kicking scenarios. For example, taekwondo athletes rely on the SSC function by performing a countermovement before the explosive triple extension to generate power during sequences of successive kicks [3] or to facilitate executing more powerful jump kicks, standing, and airborne kicks [4]. Although extensive literature has reported consistently that more competitive taekwondo athletes jumped higher than less successful counterparts [2,6,8,10], this was the first study to uncover the abovementioned neuromuscular mechanisms behind better CMJ JH from the more successful athletes in the taekwondo population. 

The current study showed that CMJ ConPV was significantly greater among MG and ConPF, ConPP, and RSImod but did not reach between-group statistical differences. However, moderate to large effect sizes were observed between the two cohorts. Greater values in RSImod among the MG revealed that more successful athletes can achieve greater JH without increased TTT. A similar outcome has been reported from a previous study, suggesting ConPF and ConPP normalized to body mass could not distinguish between different competitive levels of rugby players [14]. However, movement velocity is essential for taekwondo [3], as the ability to produce greater velocity is a favorable physical trait for the MG in facilitating kicking techniques during sparring [33]. 

The second finding of this study revealed that the MG had superior SLLH performance to the NMG. Although none of the variables reached statistical between-group differences, the MG outperformed the NMG in all SLLH metrics with moderate effect sizes (d = 0.40–0.84). Not only was the MG able to jump farther in step 1 (*d* = 0.61), but the greater step 2 hopping distance achieved, alongside shorter GCT (*d* = 0.63) that resulted in greater values of LRSI (*d* = 0.84), also implied their better reactive strength by utilizing the SSC mechanics to potentiate the subsequent take-off after landing from step 1. 

The optimal reactive strength of the SSC function facilitates and underpins many taekwondo actions, such as the rapid hopping action to approach the opponent before launching an attack [34] and the execution of double kick techniques. Following each strike, the leg is quickly driven back down into the ground and then quickly driven back up toward the opponent [3]. Therefore, the SLLH could be an assessment tool for lateral rapid force production ability among taekwondo.

Finally, this study found that certain variables between CMJ and SLLH have moderate to high correlations (r = 0.50–0.57), which implies some shared neuromuscular characteristics in performing these different types of jump tasks among taekwondo athletes. 

Results revealed that SLLH step 1 was significantly correlated with CMJ concentric variables. The moderately significant correlation between SLLH step 1 and CMJ JH, ConPP, ConPV, and RSImod implicated SLLH’s initial force production ability, which is more linked to the concentric portion of CMJ. Like CMJ JH, depending upon the power applied vertically, the SLLH test requires the generation of leg power to maximize horizontal distance. Studies in the past also indicated that despite the difference in body positioning between vertical and horizontal jumps, one might infer that similar muscle activity occurs to produce extension at the hip, knee, and ankle joints [35]. On the other hand, the finding was consistent with previous studies indicating this pre-stretch augmentation of SSC was much less in the horizontal plane jump tasks [36], implying horizontal jump tasks are more likely to rely on the concentric muscular strength and acceleration of the lower extremity.

However, both concentric and eccentric strength seem important after step 1. Data show that SLLH GCT and LRSI represent subsequent force production ability after step 1. Correlation with F@0V represents the eccentric strength required to absorb landing forces from step 1. In contrast, ConPF is needed to initiate a subsequent take-off rapidly with minimal GCT during the dynamic coupling transition while maximizing hopping distance. Such successive combinations of eccentric and concentric muscle contractions are commonly required in athleticism [21]. 

Although similar mechanics between the CMJ and SLLH were found according to the result of correlation analysis, the SLLH reflects different neuromuscular abilities from the CMJ among taekwondo athletes. For example, taekwondo fighting scenarios involve multiplanar actions [19]; the initiation of a single kick with maximum effort is reliant on acceleration and power, while the frequent skipping motions to approach the opponent and to prepare a new attack laterally depend upon reactive strength applied vertically and laterally [3,34]. 

## 5. Conclusions

In summary, the CMJ distinguished between collegiate male taekwondo medalists and non-medalists. Notably, the study’s results illustrated better lower extremity eccentric strength and lateral power among the medalist group. Using SLLH, which matches taekwondo-specific movement patterns along CMJ, has provided new insights into the differences in neuromuscular function between two jump tasks. However, limitations of this study are noted. The sample size of this study is relatively small. Not considering weight categories and anthropometric factors as part of the research question would be regarded as a limitation associated with this study. Despite the limitations, incorporating SLLH along with CMJ can still benefit the ongoing athlete-monitoring process in the taekwondo population. Further investigation is recommended among different and extended taekwondo populations (e.g., female athletes and international competitors) in weight categories. 

Based on the results of the current study, more successful collegiate taekwondo athletes have better eccentric strength than their less successful counterparts. Therefore, enhancing eccentric capabilities among taekwondo athletes may be a competitive advantage. Training program design is consequently recommended to target the development of rapid eccentric strength with exercises such as accentuated eccentric loading training, loaded jumps, high-intensity plyometrics, etc. Furthermore, because repeated lateral rapid expressions of force are essential in taekwondo, strength training interventions should develop SSC mechanisms in the lateral direction (e.g., lateral hurdle hops or jumps) and be periodized appropriately according to the competition schedule.

## Figures and Tables

**Table 1:** Countermovement jump (CMJ) variable descriptions.

CMJ Variable	Abbreviation	Description
Jump height (cm)	JH	Maximum JH (calculated using the velocity at take-off method)
Time to take-off (s)	TTT	Time spent from the onset threshold to take-off
Reactive strength index modified	RSImod	Jump height divided by TTT
**Eccentric phase variables**		
Peak eccentric force (N·kg^−1^)	EccPF	Maximum force value attained during the eccentric phase
Force at 0 velocity (N·kg^−1^)	F@0V	Force exerted at the end of the countermovement (i.e., velocity is at zero)
Peak eccentric power (W·kg^−1^)	EccPP	Maximum power value attained during the eccentric phase
Peak eccentric velocity (m·s^−1^)	EccPV	Minimum velocity attained during the eccentric phase
Eccentric displacement (cm)	EccDis	Lowest point of the countermovement
**Concentric phase variables**		
Peak concentric force (N·kg^−1^)	ConPF	Maximum force value attained during the concentric phase
Peak concentric power (W·kg^−1^)	ConPP	Maximum power value attained during the concentric phase
Peak concentric velocity (m·s^−1^)	ConPV	Maximum velocity attained during the concentric phase

**Table 2:** Comparison of CMJ variables between MG and NMG.

	MG (n = 8)	NMG (n = 9)	*p*	*d*	Magnitude Descriptor	ICC (95% CI)	% CV
CMJ Variables	Mean ± SD	Mean ± SD					
JH (cm)	44.8 ± 5.18	38.3 ± 3.95	0.01	1.40	Large	0.978 (0.942–0.992)	0.57
TTT (s)	0.84 ± 0.47	0.84 ± 0.12	0.77	0.02	Trivial	0.921 (0.795–0.971)	2.28
RSImod	0.54 ± 0.06	0.47 ± 0.07	0.06	1.03	Moderate	0.951 (0.869–0.982)	2.8
**Eccentric phase variables**							
EccPF (N·kg^−1^)	26.12 ± 2.65	23.07 ± 1.74	0.04	1.36	Large	0.928 (0.814–0.973)	2.14
F@0V (N·kg^−1^)	26.05 ± 2.57	23.69 ± 1.72	0.04	1.08	Moderate	0.929 (0.815–0.974)	2.04
EccPP (W·kg^−1^)	−22.76 ± 5.51	−19.15 ± 5.14	0.18	−0.68	Trivial	0.933 (0.826–0.975)	5.57
EccPV (m·s^−1^)	−1.54 ± 0.22	−1.37 ± 0.25	0.14	0.72	Moderate	0.938 (0.840–0.977)	3.35
EccDis (cm)	−41.57 ± 6.35	−35.07 ± 5.99	0.05	1.05	Moderate	0.945 (0.842–0.981)	3.26
**Concentric phase variables**							
ConPF (N·kg^−1^)	26.21 ± 2.54	24.27 ± 1.62	0.09	0.91	Moderate	0.935 (0.829–0.976)	1.81
ConPP (W·kg^−1^)	59.02 ± 6.07	54.73 ± 4.18	0.11	0.82	Moderate	0.970 (0.920–0.989)	1.23
ConPV (m·s^−1^)	3.05 ± 0.17	2.85 ± 0.13	0.01	1.32	Large	0.982 (0.952–0.993)	0.65

Abbreviations: MG = medalist group; NMG = non-medalist group; JH = jump height; TTT = time to take-off; RSImod = reactive strength index modified; F@0V = force at 0 velocity; EccPF = eccentric peak force; EccPP = eccentric peak power; EccPV = eccentric peak velocity; EccDis = eccentric displacement; ConPF (N·kg^−1^) = concentric peak force; ConPP (W·kg^−1^) = concentric peak power; ConPV (m·s^−1^) = concentric peak velocity.

**Table 3:** Comparison of SLLH variables between MG and NMG.

	MG (n = 8)	NMG (n = 9)	*p*	*d*	Magnitude Descriptor
SLLH Variables	Mean ± SD	Mean ± SD			
Step 1 (cm)	169.63 ± 19.86	159.11 ± 13.00	0.21	0.61	Moderate
Step 2 (cm)	246.38 ± 31.36	235.67 ± 20.64	0.41	0.40	Small
TotalD (cm)	416.00 ± 49.17	394.78 ± 30.91	0.30	0.52	Small
GCT (s)	0.27 ± 0.02	0.29 ± 0.04	0.14	0.63	Moderate
LRSI	908.97 ± 116.20	811.36 ± 114.43	0.10	0.84	Moderate

Abbreviations: MG = medalist group; NMG = non-medalist group; step 1 = distance from start line to the first landing; step 2 = distance from the first landing to the second landing; TotalD = summation distance of step1 and step 2; GCT = ground contact time; LRSI = lateral reactive strength index.

**Table 4:** Pearson’s correlations (*r*) among CMJ and SLLH selected variables.

Variable	CMJ JH	CMJ RSImod	CMJ EccDis	CMJ TTT	CMJ EccPF	CMJ ConPF	CMJ F@0V	CMJ EccPP	CMJ ConPP	CMJ EccPV	CMJ ConPV
SLLH Step 1	**0.565**	**0.495**	−0.268	−0.044	0.155	0.203	0.150	0.022	**0.497**	−0.080	**0.544**
SLLH Step 2	0.305	0.370	−0.066	−0.197	0.253	0.268	0.257	−0.016	0.186	−0.020	0.267
SLLH TotalD	0.430	0.442	−0.153	−0.144	0.226	0.255	0.227	−0.001	0.326	−0.046	0.397
SLLH GCT	−0.065	−0.211	0.009	0.255	**−0.541**	**−0.532**	**−0.555**	0.234	−0.195	0.158	−0.098
SLLH FT	0.060	0.079	−0.117	−0.073	0.134	0.038	0.136	−0.169	−0.044	−0.154	0.032
SLLH LRSI	0.278	0.449	−0.039	−0.357	**0.562**	**0.593**	**0.577**	−0.142	0.298	−0.101	0.272

Abbreviations: JH = jump height; TTT =time to take-off; RSImod = reactive strength index modified; force at 0 velocity; EccPF = eccentric peak force; EccPP = eccentric peak power; EccPV = eccentric peak velocity; EccDis = eccentric displacement; ConPF (N·kg^−1^) = concentric peak force; ConPP (W·kg^−1^) = concentric peak power; ConPV (m·s^−1^) = concentric peak velocity; step 1 = distance from start line to the first landing; step 2 = distance from the first landing to the second landing; TotalD= summation distance of step 1 and step 2; GCT = ground contact time from step 1 to step 2. Note: Bold values indicate a significant correlation (*p* < 0.05).

## Data Availability

The original contributions presented in this study are included in the article; further inquiries can be directed at the corresponding author.
